# Ambulatory Laparoscopic Appendectomy: Does the Conventional Approach Need a Reappraisal?

**DOI:** 10.7759/cureus.29215

**Published:** 2022-09-15

**Authors:** Ismail Aydin, Ilker Sengul, Mert Gungor, Tugrul Kesicioglu, Demet Sengul, Selahattin Vural, Elmas Yimaz

**Affiliations:** 1 General Surgery, Giresun University Faculty of Medicine, Giresun, TUR; 2 Endocrine Surgery/General Surgery, Giresun University Faculty of Medicine, Giresun, TUR; 3 Pathology, Giresun University Faculty of Medicine, Giresun, TUR

**Keywords:** pathology, surgical pathology, histopathology, emergency, ambulatory surgery, laparoscopic appendectomy, laparoscopy, appendectomy, acute appendicitis, acute abdomen

## Abstract

*A Deucalione*, acute abdomen remains significant in abdominal pain. The entity of acute abdomen accounts for up to 10% of all emergency admissions. The differences between countries' income and level of prosperity are pertinent, particularly in terms of severity, radiological modalities, and surgical management of the condition. Of note, surgical modalities have been the most widely used treatment modality, and current evidence indicates that the laparoscopic approach, *per se*, is the most effective surgical therapy with a lower incidence of wound infection, post-intervention morbidity, shorter hospital stay, and better quality of life scores compared to the conventional method. In light of this, the present study aimed to evaluate ambulatory appendectomy in a series of sequential laparoscopic appendectomies (LApp), which included both complicated and uncomplicated cases.

## Introduction

Acute abdominal pain accounts for 7-10% of all ED admissions. Acute appendicitis (AA), *per se*, is among the most common causes of acute abdominal pain and is the most common diagnosis in young patients admitted to the hospital with acute abdomen. The incidence rate of AA ranges from 5.7 to 50 per 100,000 people per year in developed countries and peaks between the ages of 10-30 years [1-3]. The lifetime risk for AA is reported to be 9% in the United States (US), 8% in Europe, and 2% in Africa. In addition, there are great differences in the severity, radiological examinations, and surgical management of AA depending on the country's income levels [4,5].

*A posteriori*, surgery has been the most widely accepted treatment modality, with more than 300,000 appendectomies performed each year in the US. Of note, current evidence indicates that laparoscopic appendectomy (LApp) is the most effective surgical treatment, associated with a lower incidence of wound infection and post-intervention morbidity, shorter hospital stay, and better quality of life scores compared to conventional appendectomy (CApp) [6-8]. Despite all the advances in the diagnostic process for AA disease, difficulties still remain in terms of the indication for surgery. In recent years, there has been a renewed interest due to the increase in minimally invasive techniques, safer postoperative complication rates, and early discharge rates [9-11]. Also, in recent years, appendectomy has been increasingly performed as "short stay" (<24-hour hospital stay) or "ambulatory" (no overnight hospital stay) [12-19] procedures with satisfactory results and low complication rates [19-22]. Still, traditional hospitalization, with an overnight stay, remains the routine procedure for patients with AA.

Of note, the percentage of appendectomy procedures performed in ambulatory settings that have been reported in the literature varies widely, ranging from 20 to 88% [12-22], possibly due to inconsistent definitions of ambulatory surgery (AmbSurg) and the presence of different criteria for recording ambulatory data. There is no current consensus on the selection of patients with acute AA for ambulatory (outpatient, Ambsurg) surgery. AmbSurg, *per se*, is associated with up to less than 12 hours of hospital stay without overnight hospitalization.

The present study aimed to evaluate a series of sequential LApps, both complicated and uncomplicated, performed at our center and to determine the safety of ambulatory appendectomy. In addition, the secondary aim was to explore factors associated with unexpected re-consultations and readmissions, with the objective of arriving at findings that could improve patient selection for ambulatory appendectomy.

## Materials and methods

The documents and the relevant data of 69 patients who underwent laparoscopic surgery with the diagnosis of AA in the Ministry of Health-Giresun University Training and Research Hospital from January 2018 to December 2019 were retrospectively analyzed and incorporated into the study. Parameters such as age, sex, body mass index (BMI), histopathology, American Society of Anesthesiologists (ASA) score, Saint-Antoine score, appendix diameter, white blood cell (WBC) count, neutrophil, complaint time, surgery time, drain application, closure of the appendix stump, diet, complications, re-hospitalizations, and redo surgery were evaluated between the cases with hospitalization time <24 hours and those with ≥24 hours. The statistical analysis was performed with MedCalc (MedCalc Software, Mariakerke, Belgium). The conformity of continuous variables to normal distribution was investigated by the Kolmogorov-Smirnov test. The variables with Gaussian distribution were presented as mean ±SD, while those with non-gaussian distribution were shown as median (25th percentile-75th percentile). The Student's t-test was used for intergroup comparisons of the variables with normal distribution, and the Mann-Whitney U test was used for intergroup comparisons of the variables with non-normal distribution. Pearson's chi-squared test or Yates' chi-squared test was used to compare group ratios, and the evaluation of the diagnostic performance of Saint-Antoine scoring was done with receiver operating characteristic (ROC) analysis.

## Results

The demographic, clinical, and laboratory data of the entire study population are summarized in Table 1. The mean value of the appendix diameter and the median C-reactive protein (CRP) value were statistically significantly lower in the group with a hospitalization duration of <24 hours compared to the group with a hospital stay longer than ≥24 hours for day discharge (p=0.008 and p<0.0001, respectively); the number of patients with a Saint-Antoine score ≥3 was also statistically significant (p=0.005). In addition, the percentage of patients using drains in the group who stayed in the hospital for ≥24 hours, the percentage of patients starting the regimen and peroral nutrition on the second or third day, and the percentage of endo-loop application as a form of appendiceal stump closure were statistically significant (p=0.020 and p<0.000, respectively). The optimal cutoff values ​​and sensitivity and specificity values ​​were determined by ROC analysis, where the diagnostic performances of the four numerical variables were included in the Saint-Antoine score, which defined the group with <24 hours for the hospital stay, 26.5 kg/m^2^ for BMI (sensitivity: 61.0%; specificity: 60.7%), 15.2 x 10^3^/µL for WBC (sensitivity: 80.5%; specificity: 60.7%), 217 mg/L for CRP (sensitivity: 95.1%; specificity: 67.9%), 10.3 mm for appendix diameter (sensitivity: 82.9%; specificity: 64.3%) (Table 2). The cutoff values ​​for these parameters were consistent with values ​​classically defined in the Saint-Antoine score, excluding CRP (28 kg/m^2^, 15 x 10^3^/µL, 30 mg/L, and 10 mm for BMI, WBC, CRP, and appendix diameter, respectively).

**Table 1:** Demographic, clinical, and laboratory data of the study population (n=69) SD: standard deviation; BMI: body mass index; ASA: American Society of Anesthesiologists

Variables	Values
Age, years, mean ±SD	37.3 ±14.3
Gender, M/F	44/25
BMI, kg/m^2^, mean ±SD	27.3 ±6.0
Histopathology	
Acute appendicitis, n (%)	60 (87.0%)
Perforated appendix, n (%)	7 (10.1%)
Necrotic appendix, n (%)	2 (2.9%)
ASA score	
1, n (%)	49 (71.0%)
2, n (%)	19 (27.5%)
3, n (%)	1 (1.4%)
Saint-Antoine score	
0, n (%)	1 (1.4%)
1, n (%)	10 (14.5%)
2, n (%)	22 (31.9%)
3, n (%)	22 (31.9%)
4, n (%)	22 (31.9%)
5, n (%)	4 (5.8%)
Apendiks diamater, mm, mean ±SD	10.7 ±5.7
WBC, x 10^3^/µL, mean ±SD	13.7 ±4.0
Neutrophils, x 10^3^/µL, mean ±SD	10.9 ±4.0
CRP, mg/L, median (25th percentile-75th percentile)	132 (54-248)
Complaint time, hours, median (25th percentile-75th percentile)	48 (14-72)
Operation time, minutes, mean ±SD	60.1 ±19.3
Drain application	
None, n (%)	52 (75.4%)
Yes, n (%)	17 (24.6%)
Closure of the appendix stump	
Hemoclip + hemoloc, n (%)	46 (66.7%)
Endo-loop, n (%)	21 (30.4%)
Intercorporeal, n (%)	2 (2.9%)
Hospitalization, hours, median (25th percentile-75th percentile)	24 (24-48)
Diet	
1stday, n (%)	59 (85.5%)
2ndday, n (%)	9 (13.0%)
3rdday, n (%)	1 (1.4%)
Complication	
None, n (%)	64 (92.8%)
Wound infection, n (%)	2 (2.9%)
Hernia, n (%)	1 (1.4%)
Fever, n (%)	1 (1.4%)
Re-hospitalization	
None, n (%)	67 (97.1%)
Yes, n (%)	2 (2.9%)
Redo surgery	
None, n (%)	68 (98.6%)
Yes, n (%)	1 (1.4%)

**Table 2:** ROC analysis results* *Evaluating the diagnostic performance of four numerical variables defined in the Saint-Antoine score for ambulatory discharge ROC: receiver operating characteristic; AUC: area under the curve; BMI: body mass index; WBC: white blood cells; CRP: C-reactive protein

Variable	AUC (95% CI)	Cutoff	Sensitivity	Specificity
BMI	0.580 (0.443-0.716)	26.5 kg/m^2^	61.0%	60.7%
WBC	0.631 (0.482-0.780)	15.2 x 10^3^/µL	80.5%	60.7%
CRP	0.815 (0.699-0.931)	217 mg/L	95.1%	67.9%
Appendix diameter	0.689 (0.554-0.824)	10.3 mm	82.9%	64.3%

**Figure 1:**
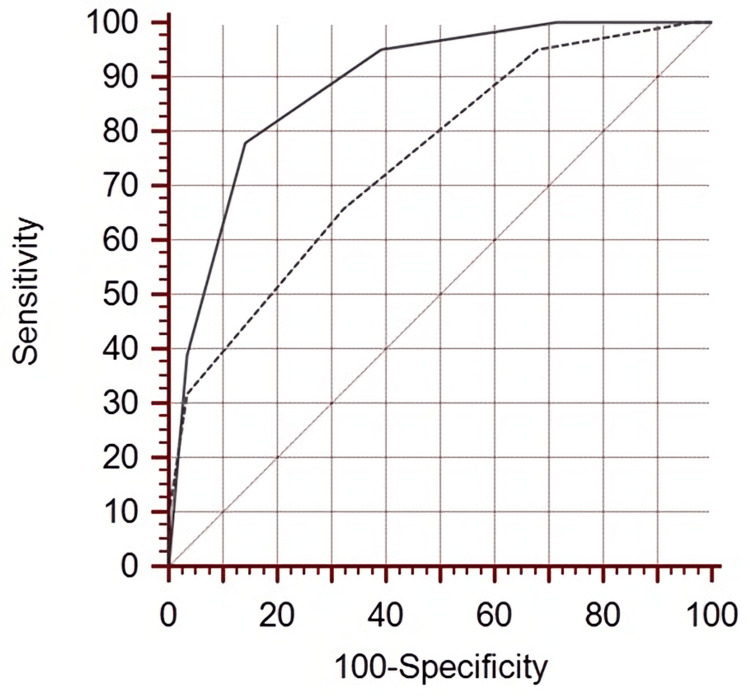
ROC graph* *Evaluating the diagnostic performance of the classical Saint-Antoine score (dashed line) and the revised Saint-Antoine score (solid line) for day discharge ROC: receiver operating characteristic

The area under the curve (AUC) value was calculated as 0.749 (95% CI: 0.634-0.864) by ROC analysis, in which the diagnostic performances of the classical Saint-Antoine score were evaluated for day discharge. The cutoff value with the most appropriate sensitivity and specificity value was ≥3 (sensitivity: 65.9%; specificity: 67.9%). In addition, considering the cutoff values ​​specified in Table 3 for the numerical variables of this score, the AUC value for the diagnostic performance of the revised Saint-Antoine score was calculated as 0.885 (95% CI: 0.803-0.968), The cutoff value with the most appropriate sensitivity and specificity value was ≥4 (sensitivity: 78.1%; specificity: 85.7%) (Figure 1). The Saint-Antoine Score was used to determine the severity of appendicitis in the present study (Table 4).

**Table 3:** Saint-Antoine scores for day discharge and the cutoff values ​​for their numerical variables

	Classical Saint-Antoine score		Revised Saint-Antoine score	
Criterion	Sensitivity (95% CI)	Specificity (95% CI)	Sensitivity (95% CI)	Specificity (95% CI)
≥2	95.1% (83.4%-99.3%)	32.1% (15.9%-52.3%)	100% (91.3%-100%)	28.6% (13.3%-48.7%)
≥3	65.9 (49.4%-79.9%)	67.9% (47.7%-84.1%)	95.1% (83.4%-99.3%)	60.7% (40.6%-78.5%)
≥4	31.7% (18.1%-48.1%)	96.4% (81.6%-99.4%)	78.1% (62.4%-89.4%)	85.7% (67.3%-95.9%)
≥5	9.8% (2.8%-23.1%)	100% (87.5%-100%)	39.0% (24.2%-55.5%)	96.4% (81.6%-99.4%)

**Table 4:** Saint-Antoine score BMI: body mass index; WBC: white blood cells; CRP: C-reactive protein

Parameters	Saint-Antoine score
BMI <28 kg/m^2^	1
WBC <15,000/μL	1
CRP <3 mg/dL	1
Radiological imaging without evidence of perforation	1
Appendix diameter ≤10 mm	1

## Discussion

Globally, AA is the most common emergency surgical disease with a lifetime incidence rate of 8.6% for men and 6.7% for women [18,22]. Despite the availability of antibiotic therapy as a non-invasive alternative, LApp remains the standard treatment for AA [10]. Some authors have used Saint-Antoine scoring for severity assessment in their study [12]. Sabbagh et al. [18], in a prospective study, showed that the rate of hospital stay of <24 hours after LApp could be as high as 52%. This rate rises to 73% after excluding patients with complicated appendicitis who have medical or social contraindications to the ambulatory procedure.

Cash et al. [19] have reported that the augmentation of the same-day discharge success rate ranges from 35% to 85% for uncomplicated AA without an increase in morbidity (8.4% vs. 5.2%). AmbSurg is defined as a hospital stay of <12 hours, which indicates that LApp should be performed during the daytime so that surgery is usually not delayed till the next day and the patient should return home the same night. However, hospitalization may be required on the same night, depending on the patient's time of admission to the hospital and the availability of the operating room during the day. Some retrospective studies have demonstrated the feasibility of LApp for AA during a hospital stay of <24 hours. Alverez et al. [13] reported that 57% of patients were discharged within the first 24 hours of the treatment for acute perforated appendicitis. Gilliam et al. [15] reported that of the 104 LApp cases, nine were discharged within the first eight hours and 66 patients were discharged within the first 24 hours. Frazee et al. [21] reported that 88% of 348 non-complicated appendicitis patients were discharged on the same day after LApp and postoperative morbidity was observed in 6.6% of the patients while re-hospitalization was observed at a rate of 1%. Schreiber [23] reported on 78 female patients and concluded that ambulatory LApp is a safe and cost-effective method in outpatient conditions for the treatment of acute and subacute appendicitis in selected cases, compared with conventional therapy, CApp.

Cross and Kowdley [24] reported that 55 of 84 cases were discharged in less than 24 hours and an average of 25 hours was observed in their study, with a minimum stay of two hours and a maximum stay of 96 hours. One of the cases, who was discharged in <24 hours, required re-hospitalization. Histopathology revealed appendicitis in 52 (95%) cases and a total of 19 cases (22%) were discharged from the recovery room in <7 hours. In addition, no readmission was reported, and there was no significant difference in terms of complications or readmission between patients who were discharged in <24 hours and those who stayed longer. de Wijkerslooth et al. [25] concluded that same-day discharge after LApp for uncomplicated appendicitis is safe without an augmented risk of readmission, complications, or unplanned hospital visits. As a matter of fact, emergency surgery remains significant and severe globally in the mentioned era [26-30].

Limitations

Our study has some limitations. Primarily, it entailed serial procedural modalities with a limited time period and a small number of cases. Moreover, the study was retrospective in design, and we could not include any study groups of cases with a cutoff discharge time of 12 hours (<24 hours and ≥24 hours).

## Conclusions

Acute abdomen retains its vital significance in Homosapiens with abdominal pain. AA still constitutes a crucial health problem for providers. Today, the laparoscopic method is globally employed and is an advanced modality in the treatment of AA. We might postulate that the so-called ambulatory LApp concept can be selectively performed in a safe manner, with the Saint Antoine Score determined before the operation, instead of CApp. We recommend that more multidisciplinary studies be conducted so that pertinent revisions to the scoring system can be implemented.
